# Dihydropyrimidinase-Related Protein 2 Is a New Partner in the Binding between 4E-BP2 and eIF4E Related to Neuronal Death after Cerebral Ischemia

**DOI:** 10.3390/ijms24098246

**Published:** 2023-05-04

**Authors:** Emma Martínez-Alonso, Alejandro Escobar-Peso, Natalia Guerra-Pérez, Marcel Roca, Jaime Masjuan, Alberto Alcázar

**Affiliations:** 1 Department of Research, Hospital Universitario Ramón y Cajal, IRYCIS, 28034 Madrid, Spain; 2 Proteomics Unit, Hospital Universitario Ramón y Cajal, IRYCIS, 28034 Madrid, Spain; 3 Department of Genetics, Physiology and Microbiology, Faculty of Biological Sciences, Universidad Complutense de Madrid, 28040 Madrid, Spain; 4 Department of Neurology, Hospital Universitario Ramón y Cajal, IRYCIS, 28034 Madrid, Spain; 5 Department of Neurology, Facultad de Medicina, Universidad de Alcalá, 28871 Alcalá de Henares, Spain

**Keywords:** cerebral ischemia, eIF4E-binding protein, eIF4E, collapsing-response mediator protein 2, dihydropyrimidinase-related protein 2, protein phosphorylation, neuronal death, neuroprotection, translation regulation, proteomics

## Abstract

Transient cerebral ischemia induces neuronal degeneration, followed in time by secondary delayed neuronal death that is strongly correlated with a permanent inhibition of protein synthesis in vulnerable brain regions, while protein translational rates are recovered in resistant areas. In the translation-regulation initiation step, the eukaryotic initiation factor (eIF) 4E is a key player regulated by its association with eIF4E-binding proteins (4E-BPs), mostly 4E-BP2 in brain tissue. In a previous work, we identified dihydropyrimidinase-related protein 2 (DRP2) as a 4E-BP2-interacting protein. Here, using a proteomic approach in a model of transient cerebral ischemia, a detailed study of DRP2 was performed in order to address the challenge of translation restoration in vulnerable regions. In this report, several DRP2 isoforms that have a specific interaction with both 4E-BP2 and eIF4E were identified, showing significant and opposite differences in this association, and being differentially detected in resistant and vulnerable regions in response to ischemia reperfusion. Our results provide the first evidence of DRP2 isoforms as potential regulators of the 4E-BP2–eIF4E association that would have consequences in the delayed neuronal death under ischemic-reperfusion stress. The new knowledge reported here identifies DRP2 as a new target to promote neuronal survival after cerebral ischemia.

## 1. Introduction

Cerebral ischemia is a cerebrovascular disease caused by an impairment of blood flow [1]. Blood hypoperfusion of brain tissue decreases the supply of metabolic substrates and oxygen, leading to the depletion of energy and the release of toxic metabolites [2,3]. These conditions are restored on subsequent blood reperfusion, though further neuronal injury is induced due to the concurrence of reactive oxygen species [4], glutamate excitotoxicity [5], inflammatory response [6], and protein synthesis inhibition [7,8]. This brain-tissue damage is followed in time by secondary cell death in adjacent and connected areas, which is known as delayed neuronal death [9]. Protein synthesis is a highly energy-dependent cellular process and is severely inhibited during ischemia. Upon reperfusion, protein synthesis is restored in the so-called resistant brain regions, such as the cerebral cortex, whereas the translation is not recovered in selective vulnerable brain areas—such as the hippocampus *cornu ammonis* one (CA1)—, regions where delayed neuronal death is induced [10,11,12]. There is solid evidence that translation inhibition and delayed neuronal death in vulnerable regions to ischemia reperfusion (IR) are closely related [8,11,12,13,14], however, the precise mechanism of this inhibition has not been fully elucidated yet.

In translation regulation, the control of the eukaryotic initiation factor (eIF) 4F complex is a critical point at the initiation step of protein synthesis, at the level of recognition of the 5′ end of mRNA, and its recruitment to the 40S ribosomal subunit. One of the eIF4F complex components, eIF4E, is a key factor in this process, which is, in turn, regulated by eIF4E-binding proteins (4E-BPs) [15,16]. Active (hypo- or dephosphorylated) forms of 4E-BPs (4E-BP1, 4E-BP2, and 4E-BP3) specifically bind to eIF4E, competing with eIF4G factor in their union with eIF4E, preventing eIF4F complex formation and inhibiting cap-dependent translation. Therefore, 4E-BPs are considered translation repressors [17,18]. Although the phosphorylation by the protein kinase mammalian target of rapamycin (mTOR) is a well-known mechanism of regulation of 4E-BP1, which inhibits its binding to eIF4E and activates protein translation [15,16], the regulation of 4E-BP2, the predominant 4E-BP expressed in the brain [19], is not well characterized. In previous studies by our group, we demonstrated that 4E-BP2 phosphorylation—or other post-translational modifications—is not the mechanism that operates on 4E-BP2 regulation under ischemia-reperfusion stress, though it is the changes in the 4E-BP2–eIF4E association that have consequences on protein-synthesis recovery or inhibition in the resistant and vulnerable brain regions, respectively [20,21]. In our goal to determine whether the protein synthesis recovery in resistant areas could be dependent on specific modulators involved in the association/dissociation of 4E-BP2–eIF4E, using a proteomic approach we recently reported new 4E-BP2-interacting proteins, which has been the first described 4E-BP2 interactome in the cerebral cortex to date, and we compared it with that of the vulnerable CA1 region [22]. The study found seven 4E-BP2-interacting proteins with changes in their association between the resistant and vulnerable brain regions after IR (Supplementary Figure S1). Interestingly, among these 4E-BP2-interacting proteins, dihydropyrimidinase-related protein 2 (DRP2) was identified [22]. DRP2, a 64-kDa protein also named collapsin response mediator protein two (CRMP2), is a cytosolic phosphoprotein mostly expressed in the brain, that is mainly involved in axonal-growth development [23]. This protein has been extensively described in experimental models of cerebral ischemia, with changes of expression related to neurodegeneration [24,25,26,27]. Moreover, DRP2 has been detected, with increased or decreased levels, in postmortem brain samples and cerebral microdialysis from stroke patients [28,29].

With this background, we decided to elucidate if DRP2, as a 4E-BP2-interacting protein, could be related to the changes observed in the 4E-BP2–eIF4E association after IR stress. To do so, we studied DRP2 in 4E-BP2 immunoprecipitates by two-dimensional (2D) fluorescence difference in gel electrophoresis (DIGE), confirming the identification of DRP2 isoforms by matrix-assisted laser desorption/ionization-time of flight (MALDI-TOF) mass spectrometry (MS) and Western blotting in the cerebral cortex and hippocampal CA1 region, after transient cerebral ischemia. Our results report different DRP2 isoforms with differential and significant changes in their association to 4E-BP2 and eIF4E between resistant and vulnerable brain regions after IR. In addition to describing DRP2 as a novel eIF4E-interacting protein, all the experimental evidence shown here demonstrates a direct protein interaction between DRP2, 4E-BP2, and eIF4E, with a higher association in the vulnerable region to IR, suggesting DRP2 as a novel modulator of the 4EBP2–eIF4E complex in translation regulation under IR stress.

## 2. Results

### 2.1. Changes in the Levels of 4E-BP2-Interacting Protein Dihydropyrimidinase-Related Protein 2 (DRP2) in Resistant and Vulnerable Regions under Ischemia Reperfusion

**Figure 1:**
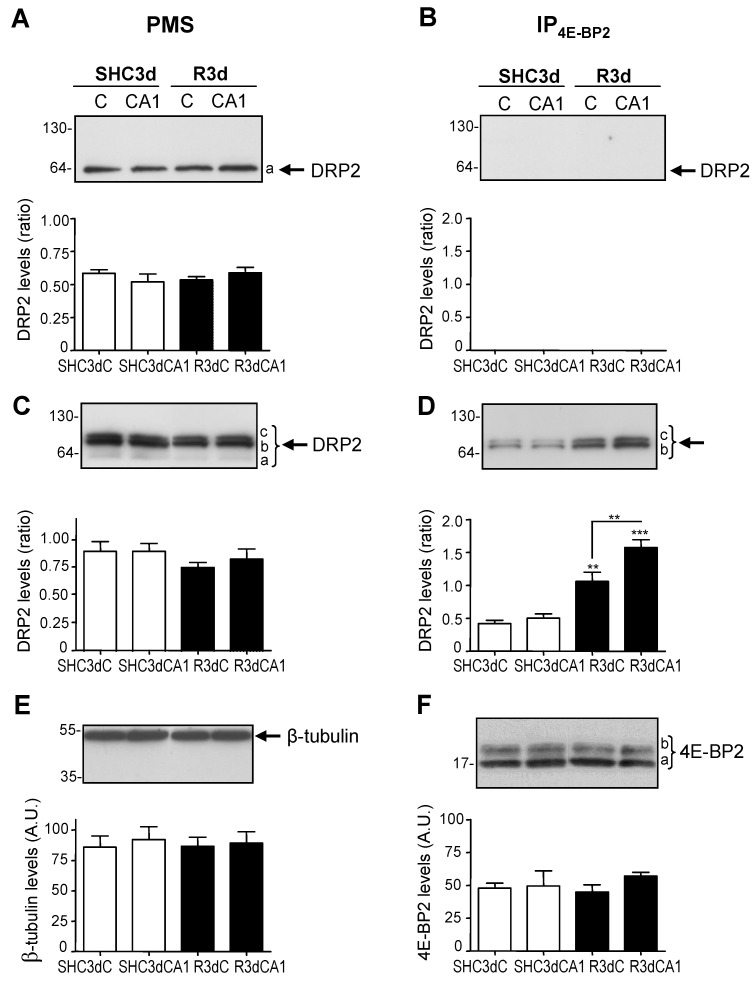
Changes in dihydropyrimidinase-related protein 2 (DRP2) levels associated with 4E-BP2. Samples (PMS) and 4E-BP2 immunoprecipitates (IP_4E-BP2_) ((**A**,**B**), respectively) of cortical (C) and hippocampal CA1 regions from control (SHC3d) and ischemic animals after 3 days of reperfusion (R3d) were analyzed by Western blotting with E9 anti-DRP2 antibody. Western blots, as in (**A**) and (**B**), were also analyzed with C16 anti-DRP2 antibody (**C**,**D**), anti-β-tubulin (**E**) or anti-4E-BP2 (**F**) antibodies, respectively. The images show representative blots, detecting DRP2 forms: *a*, *b*, and *c* (**A**–**D**); and 4E-BP2 forms: *a* and *b* (**F**). Numbers on the left of blots indicate the apparent molecular mass in kDa from protein markers. Western blot images are representative results of 4–5 independent experiments from 4–5 animals. Bar graphs under the blot images show the quantification of protein levels in the cerebral cortex and CA1 region from the control (SHC3dC and SHC3dCA1) and ischemic samples (R3dC and R3dCA1). DRP2 quantification data are with respect to 4E-BP2 levels in 4E-BP2 immunoprecipitates (ratios), or with respect to β-tubulin levels in PMS samples (ratios); data for 4E-BP2 and β-tubulin levels are expressed in arbitrary units (A.U.). Bars represent the mean of 4–5 independent experiments from 4–5 animals; error bars indicate SE. Statistical significance was determined by Newman–Keuls post-test (** *p* < 0.01; *** *p* < 0.001), after significant ANOVA, compared with their respective control, or between the cerebral cortex and CA1 samples (indicated by lines).

In our previous study of 4E-BP2-interacting proteins using a proteomic approach with 2D DIGE and MS, we described how DRP2 showed different levels of association with 4E-BP2 when comparing the cerebral cortex and hippocampal CA1 regions, in ischemic and control conditions [22]. To understand and validate these proteomic results, we analyzed DRP2 levels by Western blotting in brain samples and 4E-BP2 immunoprecipitates of cortical and CA1 regions from control (SHC3d) and ischemic animals after three days of reperfusion (R3d) (Figure 1). As a first approach, we used a monoclonal anti-DRP2 antibody (E9), which detected DRP2 in the control and ischemic samples as a single band (band *a*) in all samples (PMS) from the experimental groups (Figure 1A), though it did not recognize DRP2 in the 4E-BP2 immunoprecipitates from those samples (Figure 1B). On the other hand, a polyclonal anti-DRP2 antibody (C16) identified DRP2 in PMS samples, as well as in 4E-BP2 immunoprecipitates, detecting two bands, *b* and *c*, with lesser electrophoretic mobility—i.e., increasing the apparent molecular mass—than band *a* (Figure 1C,D). Thus, in PMS samples, three bands of DRP2 were detected: band *a* was only detected by the E9 antibody (Figure 1A); whereas bands *b* and *c* were only observed with the C16 antibody (Figure 1C); with no relevant changes in DRP2 levels between any of the experimental groups studied (Figure 1A,C). β-tubulin was used as a protein loading control in PMS and showed similar levels in all conditions (Figure 1E).

When DRP2 detection was performed on 4E-BP2 immunoprecipitates, only bands *b* and *c* were detected (Figure 1B,D), and notably, these DRP2 forms showed an increase in DRP2 association with 4E-BP2 in both cortical and CA1 regions in response to IR (Figure 1D; R3dC and R3dCA1 compared with SHC3dC and SHC3dCA1, respectively), being significantly higher in the CA1 region (R3dCA1 compared with R3dC). To assess a similar immunoprecipitation procedure in all samples, 4E-BP2 protein was analyzed as an immunoprecipitation-loading control, with the same 4E-BP2 levels subsequently observed in all the experimental samples (Figure 1F). These findings were in agreement with our results described in the proteomics study [22], that is, increased levels of DRP2 associated with 4E-BP2 in response to IR (Figure S1D; spot 3, left array), being that the association is higher in the vulnerable CA1 region than in the resistant cortical region under IR stress (Figure S1D; spot 3, right array), validating those results.

### 2.2. Multiple Isoforms of DRP2 Were Identified as 4E-BP2-Interacting Proteins

**Figure 2:**
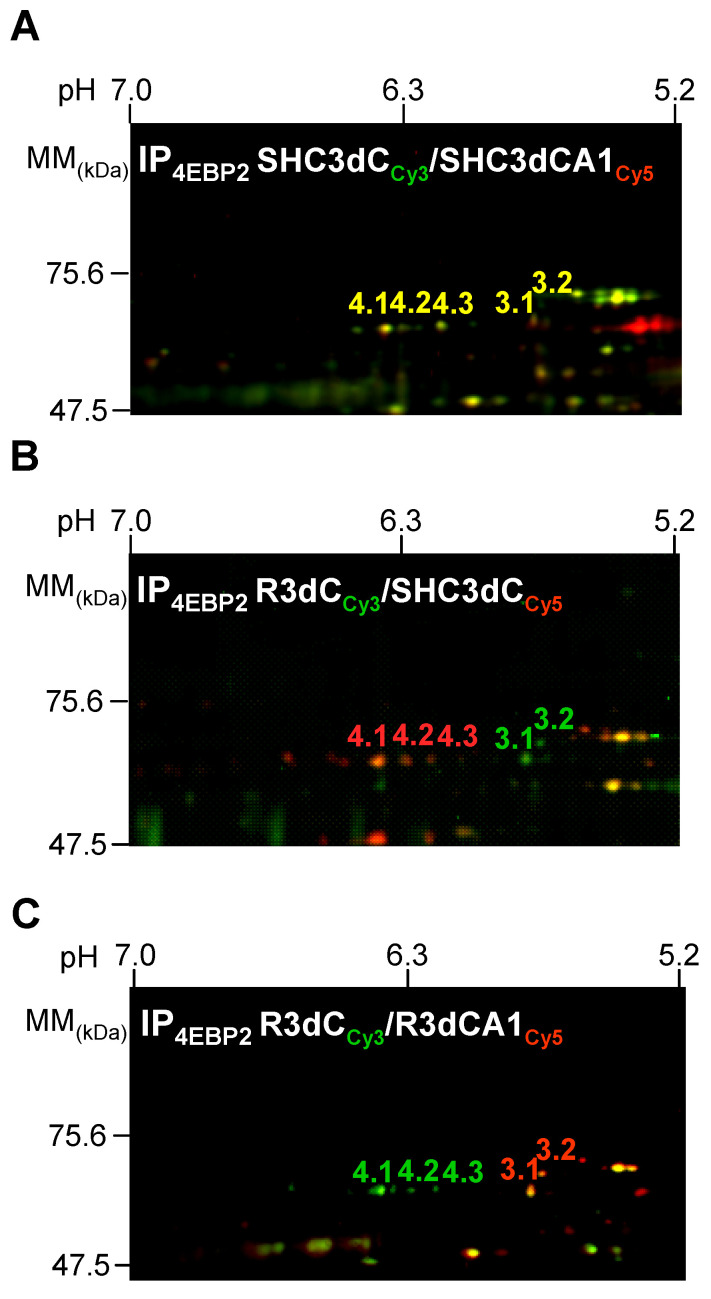
Identification of different DRP2 isoforms associated with 4E-BP2 in vivo. Two-dimensional DIGE scanned image overlays of 4E-BP2 immunoprecipitates from: (**A**), the cerebral cortex (labelled with Cy3, green) and CA1 region (labelled with Cy5, red) from control animals, (SHC3dC–SHC3dCA1 combination); (**B**), cerebral cortex samples from ischemic R3d animals (labelled with Cy3) combined with their control (labelled with Cy5), (R3dC–SHC3dC combination); and (**C**), ischemic R3d samples of the cerebral cortex (labelled with Cy3) combined with CA1 region (labelled with Cy5), (R3dC–R3dCA1 combination). The horizontal axis represents pH and the vertical axis denotes molecular mass (MM, in kDa). Images were scanned in the area of interest for DRP2 evaluation and are representative of 8 different experiments performed in paired combinations of four different samples (*n* = 4) from each of the experimental groups (SHC3dC, SHC3dCA1, R3dC, and R3dCA1), and each one of them from a pool of four independent animals. Full 2D DIGE gels are shown in the Supplementary Materials (Figure S2). DRP2 protein spots, identified by MALDI-TOF/TOF MS, were marked with numbers.

However, in the validation performed by Western blotting, we could not find any results concerning another 4E-BP2-associated DRP2 form also found in the previous 2D-DIGE experiments, with decreased DRP2 levels in response to IR (Figure S1D; spot 4). Given that DRP2 is a phosphoprotein and that multiple phosphorylated forms of DRP2 have been described [30], it is feasible that the existence of several (phospho) isoforms could explain the different DRP2 responses to IR. Then, we decided to thoroughly analyze DRP2 spots in 4E-BP2 immunoprecipitates by 2D DIGE, within the molecular mass and pI range for DRP2 (Figure 2), and its identification by MS, in order to identify whether other potential DRP2 isoforms were involved in the differential association with 4E-BP2. The identified DRP2 protein spots are shown in Table 1, along with their respective pIs and the apparent molecular mass. Therefore, five DRP2 spots were identified, where spots 3.1 and 4.1 corresponded to previously identified spots 3 and 4 of DRP2 (Figure S1D). In addition, spots labelled as 3.2, 4.2, and 4.3 in these 2D experiments were also identified as DRP2 by MS, confirming the presence of several DRP2 isoforms in the 4E-BP2 immunoprecipitates (Figure 2 and Table 1).

### 2.3. Different Isoforms of DRP2 Showed Differential Association with 4E-BP2 between Resistant and Vulnerable Brain Regions under Ischemia-Reperfusion Stress

**Figure 3:**
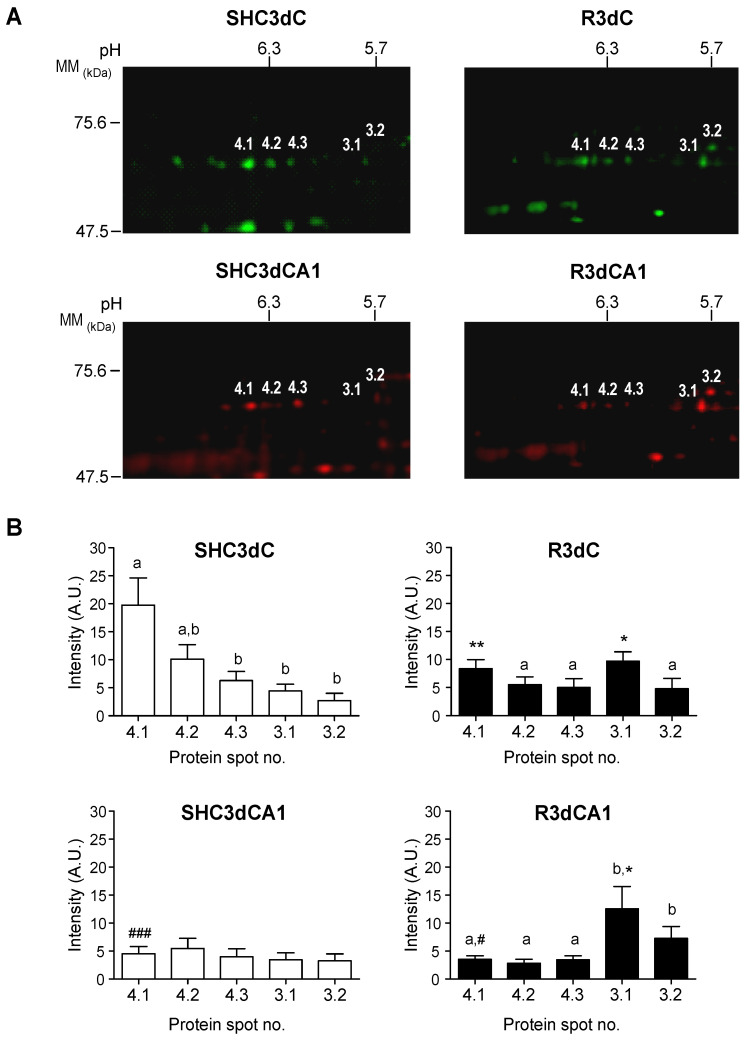
Specific induction of DRP2 isoforms interacting with 4E-BP2 in response to ischemia reperfusion. (**A**) Cropped images of the experiment shown in Figure 2 were used to quantify DRP2 isoforms. (**B**) DRP2 protein spots 3.1, 3.2, 4.1, 4.2, and 4.3 were quantified by biological variation analysis (BVA). Bar graphs show the 4E-BP2-associated DRP2 levels in control (SHC3d) and ischemic samples (R3d) of the cerebral cortex (SHC3dC and R3dC) and the CA1 region (SHC3dCA1 and R3dCA1). Data are represented as the mean of the *n* = 4 independent samples from 2D DIGE experiments. Error bars indicate SE. The vertical axis indicates the quantification values of the spot intensity in arbitrary units. Statistical significance was determined by Newman–Keuls post-test (^a^ *p* < 0.05) compared with spot 3.1, (^b^ *p* < 0.05) compared with spot 4.1, (* *p* < 0.05; ** *p* < 0.01) compared with their respective control sample, or by Student’s *t* test (^#^ *p* < 0.05; ^###^ *p* < 0.001) for comparisons between the cortical and CA1 samples, after significant ANOVA.

The quantification of 4E-BP2-associated DRP2 protein spots by biological variation analysis (BVA) in the cerebral cortex and CA1 region from control and ischemic animals, showed increased levels of DRP2 associated with 4E-BP2 in both 3.1 and 3.2 spots in IR samples (R3d) compared with the control (Figure 3). Interestingly, in the vulnerable CA1 region, this IR-induced DRP2 modification was even more marked, reaching the highest levels of the acidic isoform spot 3.1 and the lowest levels of the most basic isoform, spot 4.1 (Figure 3). Conversely, DRP2 associated with 4E-BP2 at spots 4.1, 4.2, and 4.3 showed lower levels in R3d than in the control samples, although the cerebral cortex had higher levels (statistically significant for spot 4.1) than the CA1 region (Figure 3). In addition, the increased 4E-BP2-associated DRP2 levels in the R3d ischemic condition—spots 3.1 and 3.2—were proportional to their decreased levels of DRP2 in spots 4.1, 4.2, and 4.3. Thus, the higher increase of DRP2 spot 3.1 in the CA1 region in R3d was proportional to a higher decrease of spot 4.1, in comparison with the cortical region. Remarkably, DRP2 spot 4.1 was the main DRP2 isoform in the cortical region in the control condition.

### 2.4. Identification of DRP2 Isoforms in Ischemic Samples

**Figure 4:**
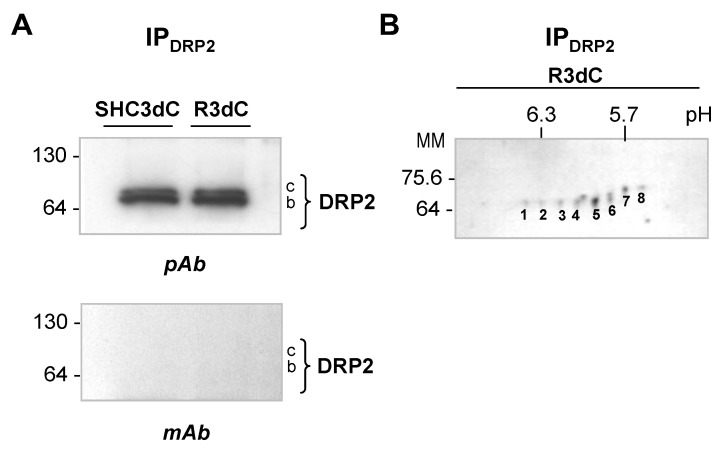
DRP2 immunoprecipitation and identification of DRP2 isoforms in ischemic samples. (**A**) Western blot of DRP2 immunoprecipitates (IP_DRP2_) from the cortical region of control and ischemic R3d samples (SHC3dC and R3dC) using C16 antibody, and developed with the same antibody (pAb), or with E6 antibody (mAb). No signal was detected with the E6 antibody. Numbers on the left indicate the apparent molecular mass in kDa from protein markers. (**B**) Two-dimensional SDS-polyacrylamide gel electrophoresis (PAGE) analysis and silver staining of immunoprecipitated DRP2. Numbers (1–8) indicate DRP2 spots/isoforms stained with a silver reagent. The horizontal axis represents pH and the vertical axis represents molecular mass (in kDa).

To identify the DRP2 isoforms observed in 4E-BP2 immunoprecipitates in the ischemic samples, the DRP2 immunoprecipitates of the cerebral cortex from the control and ischemic R3d samples were analyzed by both Western blotting and 2D SDS-polyacrylamide gel electrophoresis (PAGE), in order to confirm the correspondence between the DRP2 forms recognized by Western blotting (Figure 1) and the DRP2 spots (isoforms) identified by 2D DIGE analysis (Figure 2). First, we immunoprecipitated DRP2 using a C16 antibody and validated the immunoprecipitation by Western blotting with the same antibody. After developing the blot, two bands were detected in the DRP2 immunoprecipitates (Figure 4A), which corresponded to the DRP2 bands *b* and *c* detected in the 4E-BP2 immunoprecipitates (Figure 1D); with no presence of band *a* (Figure 4A). The 2D PAGE analysis and silver staining of immunoprecipitated DRP2 showed a total of eight DRP2 spots, numbered from one to eight, from highest to lowest pI (Figure 4B). Careful comparison of 2D PAGE and Western blot showed that spots 1–5, with a 66-kDa apparent molecular mass, corresponded to band *b*; and spots 6–8, with a 68-kDa molecular mass, corresponded to the band *c* (Figure 4). Spots 1, 2, 3, and 5 in 2D PAGE, with pI 6.4, 6.3, 6.2, and 5.8, respectively (Figure 4B), corresponded to spots 4.1, 4.2, 4.3, and 3.1, respectively, identified in 2D DIGE experiments (Figure 2 and Table 1). In addition, spot 7, with pI 5.7 (Figure 4B), corresponded to spot 3.2 in the 2D DIGE (Figure 2 and Table 1). These results have allowed the characterization of DRP2 isoforms with different pI and molecular mass, some of which were detected as associated with 4E-BP2.

### 2.5. Identification of DRP2 as an eIF4E-Interacting Protein and Its Response to Ischemia Reperfusion (IR)

**Figure 5:**
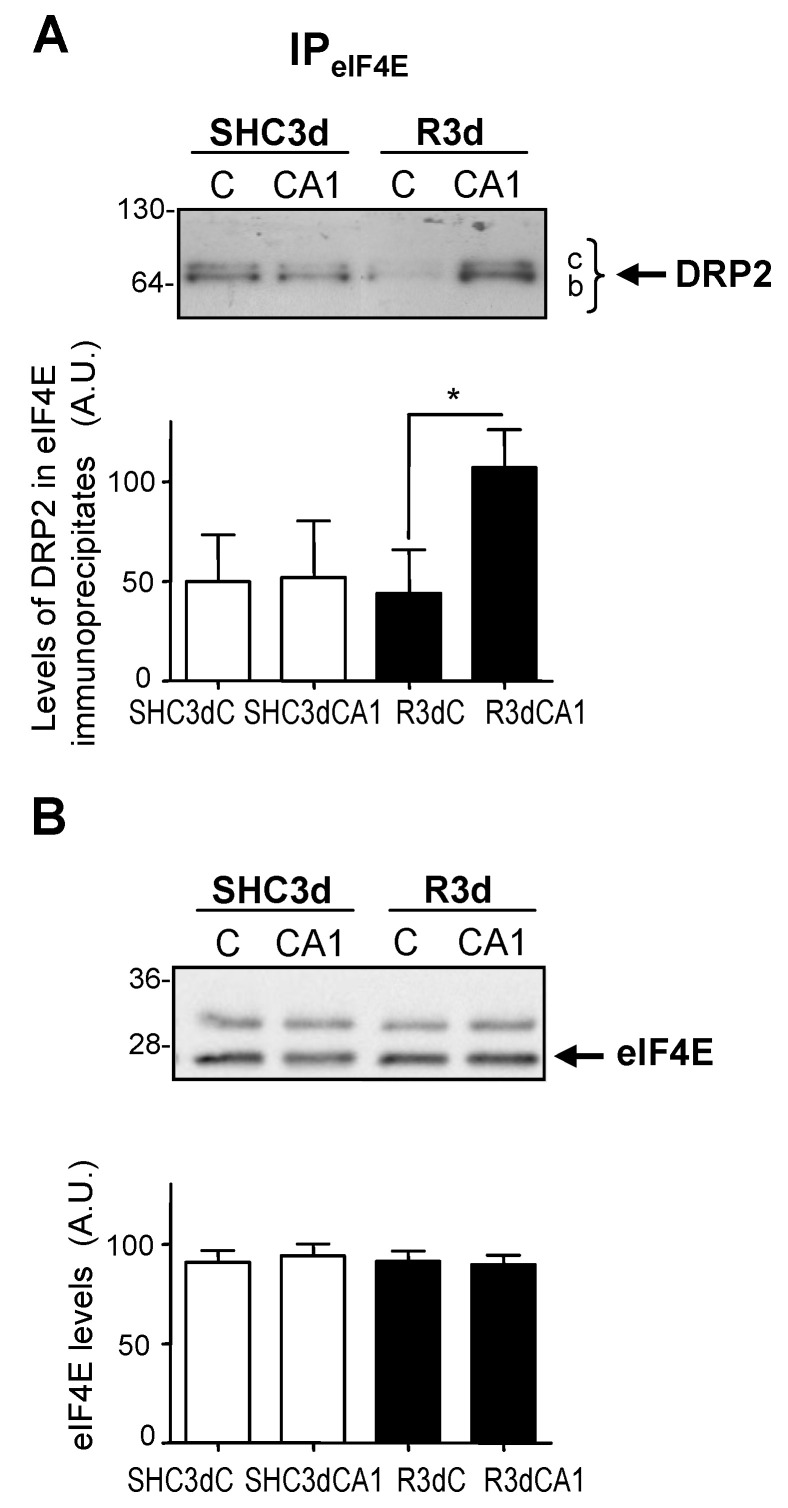
Identification of DRP2 as an eIF4E-associated protein and its response to ischemia-reperfusion stress. Samples of the cerebral cortex, C, and hippocampal CA1 region from ischemic R3d animals (R3dC and R3dCA1) and sham-control animals (SHC3dC and SHC3dCA1), were immunoprecipitated with anti-eIF4E antibody (IP_eIF4E_) and analyzed by Western blotting with C16 anti-DRP2 antibody (DRP2) (**A**), and with anti-eIF4E antibody (eIF4E) (**B**). Numbers on the left indicate the apparent molecular mass in kDa from protein markers. Bar graphs under blot images show the quantification of DRP2 and eIF4E levels in their respective Western blot and represent the mean of 3–4 independent experiments from 3–4 independent animals; error bars indicate SE. DRP2 and eIF4E levels are expressed in arbitrary units (A.U.). Statistical significance between samples was determined by Student’s *t* test (* *p* < 0.05), after significant ANOVA.

The protein 4E-BP2, as an eIF4E-binding protein [15,16], forms a complex with eIF4E. Previous results from our laboratory demonstrated an increase in the 4E-BP2–eIF4E association complex in the CA1 region in response to IR [20,21,22]. Based on these findings, and the characterization of DRP2 as a 4E-BP2-associated protein, we decided to examine the potential characterization of DRP2 as an eIF4E-interacting protein. For this purpose, we performed eIF4E immunoprecipitations and a Western blot analysis of DRP2 and eIF4E in samples of the cerebral cortex and CA1 region from control (SHC3d) and ischemic R3d animals. The results identified DRP2 in eIF4E immunoprecipitated samples, showing an interaction between DRP2 and eIF4E, being band *b* of DRP2 the main one detected by the C16 antibody (Figure 5). Interestingly, higher DRP2 levels were found in the CA1 region in the ischemic condition, having statistically significant differences in their association with eIF4E when compared to the cortical region (Figure 5A). As an immunoprecipitation loading control, the levels of immunoprecipitated eIF4E were quantified, with no differences between the assayed samples (Figure 5B). These results demonstrate a novel interaction between DRP2 and eIF4E, an association induced by IR stress in the vulnerable CA1 region. It can be inferred that the particular eIF4E-associated DRP2 isoform corresponded to spot 3.1 since it was the main DRP2 isoform present in this CA1 region upon IR condition (Figure 3B; R3dCA1) and detected at the band *b* of DRP2 (Figure 4).

**Figure 6:**
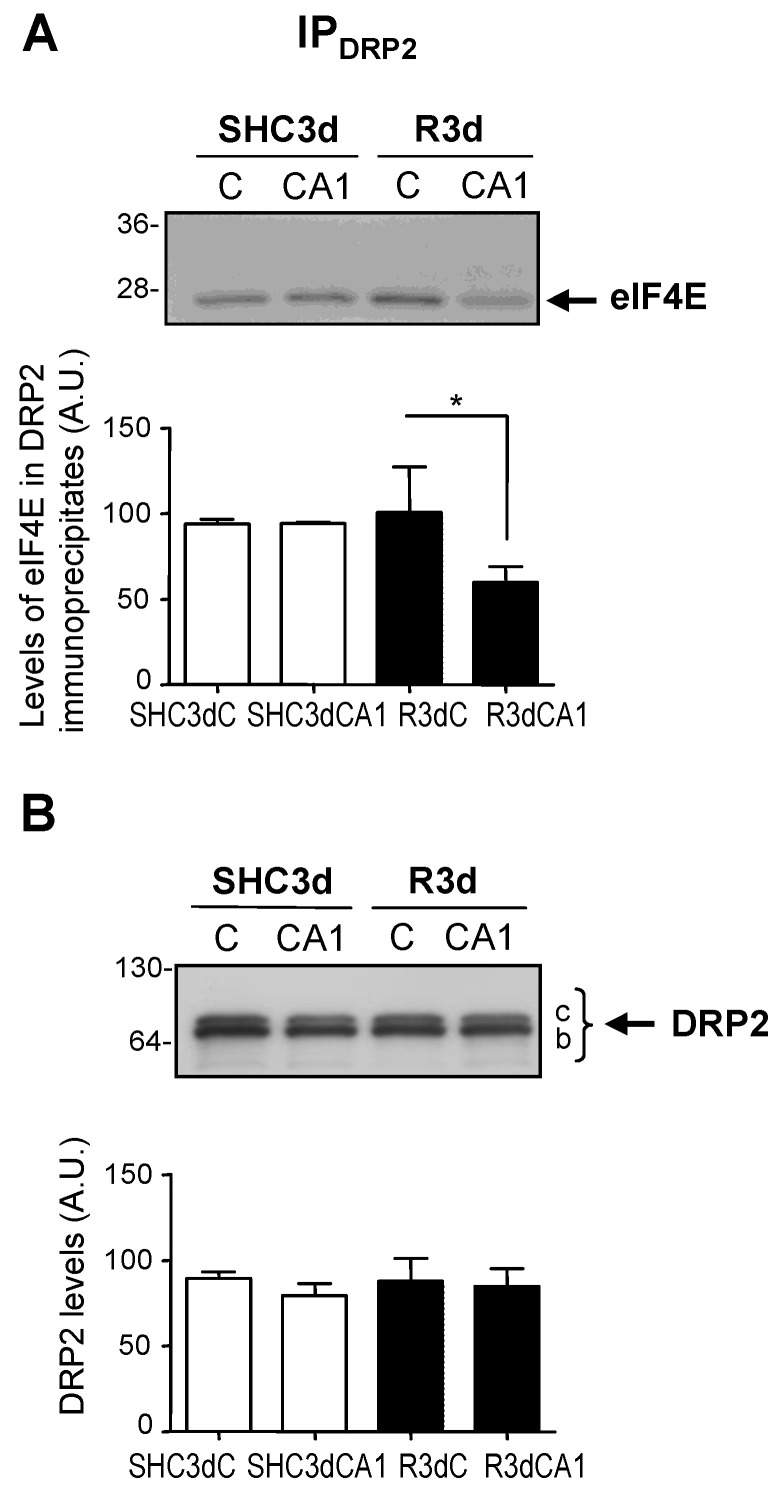
Identification of eIF4E as an interacting protein with DRP2 and its response to ischemia reperfusion. Samples, as in Figure 5, were immunoprecipitated with C16 anti-DRP2 antibody (IP_DRP2_) and analyzed by Western blotting with anti-eIF4E antibody (eIF4E) (**A**), and with C16 antibody (DRP2) (**B**). Numbers on the left indicate the molecular mass in kDa from protein markers. Bar graphs under blot images show the quantification of eIF4E and DRP2 levels in their respective Western blot and represent the mean of 3–4 independent experiments from 3–4 independent animals; error bars indicate SE. The eIF4E and DRP2 levels are expressed in arbitrary units (A.U.). Statistical significance between samples was determined by Student’s *t* test (* *p* < 0.05), after significant ANOVA.

In addition, we performed reciprocal DRP2 immunoprecipitations—with C16 anti-DRP2 antibody—to detect eIF4E and/or 4E-BP2. Interestingly, eIF4E was detected in DRP2 immunoprecipitates though, in seeming contrast to the previous results, with significantly lower levels in the CA1 region compared with the cerebral cortex in R3d ischemic samples (Figure 6A), results that we will discuss below. On the contrary, 4E-BP2 was not detected in these DRP2 immunoprecipitates. As the control of the experiments, the levels of immunoprecipitated DRP2 were quantified, without differences found between the assayed samples (Figure 6B). These results assess the specific association between eIF4E and DRP2 and confirm DRP2 as a novel eIF4E-interacting protein.

Finally, although the result of lower levels of eIF4E in the CA1 region in R3d samples could seem contradictory (Figure 6A), the fact that 4E-BP2 was not detected in DRP2 immunoprecipitates would indicate that the DRP2 associated with 4E-BP2 (and eIF4E) cannot be recognized and immunoprecipitated by the C16 antibody. Since these three proteins are jointly associated (see above results), the fraction of eIF4E associated with DRP2 and 4E-BP2 would not be detected in those DRP2 immunoprecipitates. Conversely, eIF4E associated with DRP2, in the absence of 4E-BP2, would be able to be detected in DRP2 immunoprecipitates and correspond to the results shown in Figure 6A.

## 3. Discussion

Delayed neuronal death after ischemia-reperfusion injury is strongly correlated to a permanent inhibition of protein synthesis in selectively vulnerable brain regions [8,11,12,13,14]. The contribution of the initiation step on protein translation inhibition after an ischemic episode has been well documented in numerous works reporting the formation of the ternary complex and the eIF4F complex regulation as critical steps in the control of translation inhibition associated with ischemic events [31,32,33]. In this regard, the availability of eIF4E, limited by the binding of 4E-BPs, is a key step in the control of eIF4F complex activity [15,16], though its regulation under ischemia-reperfusion stress is not yet fully known. As we previously reported, 4E-BP2-phosphorylation regulation does not operate in the permanent translation inhibition after ischemia-reperfusion stress in vulnerable brain regions—e.g., the hippocampal CA1 region—with delayed cell death [21], though it seems to be that the differential association between 4E-BP2 and eIF4E is the cause of the different susceptibility found between the vulnerable CA1 region, with increased 4E-BP2–eIF4E association and translation inhibition, in contrast to the resistant cortical region, with the lower association, and therefore higher eIF4E, available for eIF4G binding and protein synthesis recovery [20].

In the present study, we continue the recently reported research work in which we studied 4E-BP2-associated proteins in 4E-BP2 immunoprecipitates [22], in order to identify potential 4E-BP2–eIF4E-interacting proteins that could have consequences on the translation inhibition of ischemia reperfusion. Among the 4E-BP2-interacting proteins identified, DRP2 was of notable interest due to their functions in neuronal development and altered post-translational modifications in neurodegenerative disorders [34,35]. Here, using an animal model of transient cerebral ischemia and a proteomic approach with 2D DIGE combined with MALDI-TOF MS, and protein validation by Western blotting, we demonstrate the specific association of different DRP2 isoforms with 4E-BP2 and eIF4E proteins, with significant differences between resistant and vulnerable brain regions in response to ischemia reperfusion.

Different DRP2 isoforms have been described, finding up to eight DRP2 isoforms with different pI in 2D PAGE, corresponding to different phosphorylation states at multiple sites found within the carboxyl-terminal-specific region of DRP2 in vivo and in vitro [25,30]. The latter study reported the detection of three different DRP2 bands in the Western blot with low, middle, and high molecular mass (62-, 64-, and 66-kDa bands) [30], similar to the results described here, where bands of 64- (low), 66- (middle), and 68-kDa (high molecular mass) were identified and named as DRP2 bands *a*, *b*, and *c*, respectively (Figure 1). Those authors identified the band of lower molecular mass as a nonphosphorylated DRP2 form—corresponding to band *a* identified here by the E9 antibody (Figure 1A)—while bands with middle and high molecular mass were DRP2 forms with multiple phosphorylations [30]. Accordingly, we can conclude that DRP2 bands *b* and *c* detected in immunoprecipitates were phosphorylated DRP2 forms (Figure 1 and Figure 4, Figure 5 and Figure 6). Likewise, DRP2 isoforms identified in spots 4.1, 4.2, 4.3, 3.1, and spot 3.2 (Figure 2 and Figure 3), comparable to DRP2 bands *b* and *c*, respectively (Figure 4), would correspond to DRP2 with multiple phosphorylation states, increasing its phosphorylation status according to its more acidic pI.

In this study, we have characterized these DRP2 isoforms in terms of their different association with 4E-BP2 in response to ischemia reperfusion. The results showed different activity for each DRP2 isoform associated with 4E-BP2. First, the most basic isoform of DRP2 identified (spot 4.1) showed the highest association with 4E-BP2 in the cerebral cortex in control conditions, being that the more acidic isoforms are almost undetectable (Figure 3). Following postischemic reperfusion, the more acidic isoforms of DRP2 were induced, being that spot 3.1 significantly increased in parallel to a significant decrease of more basic isoforms (Figure 3), the effect that was more marked in the vulnerable CA1 region. Thus, in ischemia reperfusion, DRP2 isoforms with more acidic pI (spots 3.1 and 3.2), which could correspond to hyperphosphorylated DRP2, showed a higher association with 4E-BP2 (Figure 3) and eIF4E (Figure 5A) in the CA1 region when compared with the cerebral cortex. In contrast, in nonischemic conditions (with normal translational rates), the main DRP2 isoform with the most basic pI, spot 4.1, that could correspond to hypophosphorylated DRP2, showed a higher association with 4E-BP2 (Figure 3B) in the cerebral cortex, in which there is a lesser interaction with eIF4E (Figure 5A). These results reveal changes in the DRP2 association with 4E-BP2 and eIF4E dependent on DRP2 post-translational modifications, likely due to different phosphorylation states.

Although we did not analyze the phosphorylation status of DRP2, the close correlation with the results described by Gu et al. [30], allows us to conclude that hyperphosphorylated DRP2 would be the DRP2 form induced by ischemia reperfusion and upregulated in its association with 4E-BP2–eIF4E in the vulnerable CA1 region; while hypophosphorylated DRP2 would be the DRP2 form that participates in the association with 4E-BP2—with significant lower association with eIF4E (Figure 5)—in the control condition and the resistant cortical region in ischemia reperfusion, both situations with unrepressed protein translation.

The 4E-BP2 was not detected in the DRP2 immunoprecipitates carried out with the C16 antibody. We can assume that the C16 antibody was unable to recognize DRP2 bound to 4E-BP2 due to competition for the same DRP2 domain. It is noteworthy that the C16 antibody is developed against the carboxyl-terminal region of DRP2, the region with in vivo multiple phosphorylation sites [36]. Therefore, we can reasonably speculate that this DRP2 phosphorylation region could be the domain of the interaction with 4E-BP2, although more detailed studies will be required to confirm this hypothesis. For the same reason, eIF4E bound to 4E-BP2 would neither be immunoprecipitated nor be detected in these DRP2 immunoprecipitates, unless it was associated with DRP2 and not to 4E-BP2. Intriguingly, eIF4E was detected in the DRP2 immunoprecipitates with significantly lower levels in the CA1 region in ischemic R3d samples compared with the cerebral cortex (Figure 6A; R3dCA1), consistent with lower levels of eIF4E unbound to 4E-BP2 existing in this vulnerable region [20,21].

Several studies have reported changes in DRP2 phosphorylation related to its activity. DRP2 promotes axonal pathfinding during neural development and is involved in axonal transport and guidance, neuronal migration, and neurotransmitter release [34,35,37]. Physiologically, DRP2 is phosphorylated at Ser522 by cyclin-dependent kinase 5 (CDK5) [38], as a priming event which facilitates subsequent phosphorylation by glycogen synthase kinase 3β (GSK-3β) at Ser518, Thr514, and Thr509 [36,39,40], and by Rho-associated protein kinase (Rho-kinase) at Thr555 [41]. GSK-3β-dependent phosphorylation of DRP2 inhibits its function [36], while dephosphorylation of DRP2 by protein phosphatase 2A (PP2A) enhances axonal growth [42].

Controversial results have been described regarding DRP2 phosphorylation in brain ischemia. Dephosphorylation of DRPs has been reported in hypoxia- and ischemia-induced perinatal brain damage, although DRP2 was identified in different spots with higher phosphorylation in the hippocampus compared with the cerebral cortex [25]. DRP2 was found in human cerebral infarct samples from stroke patients, detecting DRP2 isoforms by 2D DIGE with different pI values and being downregulated or upregulated depending on the DRP2 isoform [28]. Moreover, there are other studies that found an increase in DRP2 after brain ischemia in rats [24,27]. In addition, it has been reported that there is an increase in phosphorylated DRP2 through the Akt–GSK-3β–DRP2 signalling pathway in the focal cerebral ischemia model by middle cerebral-artery occlusion (MCAO) in rats [43]. Recently, it has been reported that Toll-like receptor four (TLR4) promotes the phosphorylation of DRP2 via activation of Rho-kinase two in MCAO rats, suggesting that this mechanism may mediate the pathological outcome of TLR4 in stroke [44].

As was previously mentioned, phosphorylation of DRP2 by GSK-3β impairs its activity, leading to the inhibition of axonal/dendritic growth and neuronal activity, and inducing neuronal injury [35,36,45,46,47]. This DRP2 phosphorylation regulation has been a target for therapeutic intervention, with treatments that regulate the Akt–GSK-3β–DRP2 signalling pathway in cerebral ischemia [43,48,49]. These treatments prevented the decrease of phospho-Akt and phospho-GSK-3β levels induced by ischemia, reducing the ischemia-induced increase of phospho-DRP2 by “active” dephospho-GSK-3β, and improving the integrity and protection of axons/dendrites against ischemic injury. In addition, elevated GSK-3β activity is also associated with Alzheimer’s disease and there is a direct link with hyperphosphorylation of DRP2 at GSK-3β-dependent phosphorylation sites and increased amyloid precursor protein [37,39,45]. However, although hyperphosphorylation of DRP2 by GSK-3β and Rho-kinase proteins are associated with a reduced ability to promote axon elongation and neurite outgrowth [36,39,41], DRP2 phosphorylation by CDK5 at Ser522 seems to be necessary for a proper axonal and dendritic organization [50].

Interestingly, our results showed DRP2 markedly upregulated in reperfusion after cerebral ischemia on DRP2 isoforms with more acidic pI values, indicative of hyperphosphorylation states, reducing the potential neuronal repair in response to ischemic injury. In contrast, DRP2 isoforms with the most basic pI values, indicative of hypophosphorylated DRP2, were detected in nonischemic conditions, and also in the resistant cortical region under ischemia reperfusion, therefore being a phosphorylation status necessary for proper neuronal function.

**Figure 7:**
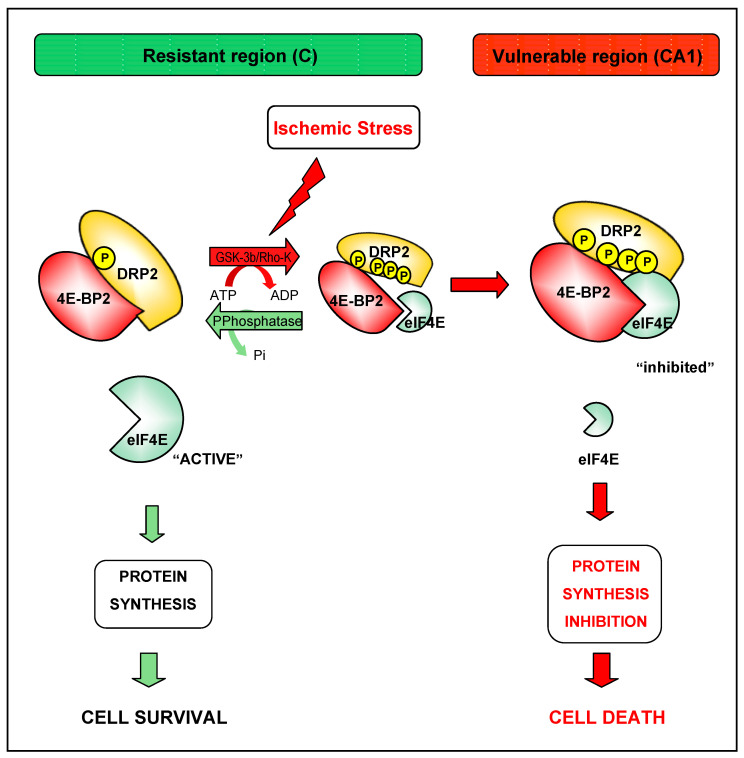
DRP2 is a key modulator of 4E-BP2–eIF4E binding in the cellular response to ischemia-reperfusion stress. Proposed model of 4E-BP2–eIF4E regulation in resistant and vulnerable regions to brain ischemia and reperfusion: 4E-BP2-interacting DRP2 protein, when it is hypophosphorylated, would compete with eIF4E, releasing this factor and promoting protein synthesis and cell protection to ischemia-reperfusion stress (e.g., in the cerebral cortex, C). Conversely, ischemia reperfusion would induce DRP2 hyperphosphorylation (possibly via GSK-3β and/or Rho-kinase) allowing the association between 4E-BP2 and eIF4E with subsequent protein-synthesis inhibition and cell-death induction.

We previously described an increase of the 4E-BP2–eIF4E complex and a decrease of the eIF4G–eIF4E “active” complex in the CA1 region compared with the cerebral cortex in cerebral ischemia [20,21] as the potential cause of the different susceptibility of these regions against ischemic-reperfusion injury. Here, we described DRP2 as a 4E-BP2-interacting protein that also binds eIF4E in nonischemic and ischemic brain samples. DRP2 hyperphosphorylation was induced by ischemia-reperfusion stress and showed, in parallel, an increased association with 4E-BP2 and eIF4E in the vulnerable CA1 region, whereas the resistant cortical region had both lower levels of this hyperphosphorylated DRP2 form—with increased hypophosphorylated DRP2—and eIF4E association. Furthermore, nonischemic brain samples showed hypophospho-DRP2 as the main form of DRP2 and also less association with eIF4E. Therefore, we can conclude an association between DRP2–4E-BP2–eIF4E in the vulnerable CA1 region after ischemia reperfusion that would reduce the availability of “active” eIF4E (unbound to 4E-BP2), essential to protein synthesis, an association that would be dependent on DRP2 phosphorylation. Thus, we propose a model in which hypophosphorylated DRP2 (the most basic pI isoform) would be bound to 4E-BP2, competing with eIF4E and releasing free “active” eIF4E in the absence of 4E-BP2 phosphorylation, allowing protein synthesis in normal conditions. In ischemia and reperfusion, DRP2 phosphorylation would be induced, yielding hyperphosphorylated DRP2 (more acidic pI isoforms) which, due to a feasible conformational change, would allow 4E-BP2 to bind eIF4E in a complex with DRP2. The latter would result in eIF4E inhibition and an arrest of translational rates in vulnerable regions with delayed neuronal death (Figure 7). However, in the resistant cortical region to ischemia, a lower increase in DRP2 hyperphosphorylation induced by ischemia reperfusion would result in adequate levels of hyperphosphorylated DRP2 associated with 4E-BP2, releasing eIF4E and inducing protein-synthesis recovery and neuronal survival in this resistant region (Figure 7).

## 4. Materials and Methods

### 4.1. Materials

Antibodies: rabbit polyclonal anti-4E-BP2 (E6532) and mouse monoclonal anti-β-tubulin (T5201) antibodies were obtained from Sigma-Aldrich (Merck KGaA, Darmstadt, Germany). Rabbit polyclonal anti-4E-BP2 antibody (#2845) was provided by Cell Signalling Technology (Beverly, MA, USA). Goat polyclonal anti-DRP2 (C16, sc-25895) and mouse monoclonal anti-DRP2 (E9, sc-376739) antibodies were purchased from Santa Cruz Biotechnology (Santa Cruz, CA, USA). Mouse monoclonal anti-eIF4E antibody (G10269) was obtained from BD Transduction Laboratories (BD Biosciences, Erembodegen, Belgium). Antimouse and antirabbit IgG peroxidase conjugated were from Cytiva (formerly GE Healthcare, Barcelona, Spain). Antigoat IgG peroxidase conjugated was from Santa Cruz Biotechnology (Santa Cruz, CA, USA). All chemicals used in isoelectric focusing and SDS-PAGE were purchased from Bio-Rad (Madrid, Spain) and Cytiva (Barcelona, Spain). All general chemicals were obtained from Sigma-Aldrich unless indicated otherwise.

### 4.2. Animal Model of Cerebral Ischemia and Reperfusion

Transient forebrain ischemia was induced in adult male Wistar rats (10–12 weeks of age, Charles River) using the standard four-vessel occlusion model described previously [31,33]. Briefly, on day 1, animals under anaesthesia (0.25 mg/kg atropine, 62.5 mg/kg ketamine, 5 mg/kg diazepam, by intraperitoneal injection) were placed in a stereotaxic frame and both vertebral arteries were permanently occluded by electrocoagulation. The following day, animals were anaesthetized (4% isoflurane sedation for induction and 2–2.5% isoflurane for maintenance in 80% N_2_/20% O_2_) during the dissection of the common carotid arteries and then both carotid arteries were occluded with atraumatic vascular clamps to induce cerebral ischemia. A neurological evaluation (corneal and righting reflex, bilateral paw extension) was performed to verify the severity of ischemia. The body temperature of the animals was monitored and maintained at 37 °C during the surgical procedure. After 15 min of arterial occlusion, clamps were removed for blood reperfusion for 3 days (R3d), and then animals were sacrificed. The cerebral cortex and hippocampal *cornu ammonis* 1 (CA1) region were dissected and immediately frozen at −80 °C. The same surgical procedures were used in sham control (SHC3d) animals except that carotid arteries were not occluded. We performed a power analysis (http://www.biomath.info/power/ttest.htm, accessed on 23 June 2020) to determine the sample size. Significance level and statistical power were set at 0.05 and 0.8 (80%), respectively, which afforded a sample size of <6 subjects per group. Animals, 48 in total, were divided into 24 sham control and 24 ischemic R3d animals.

### 4.3. Brain-Tissue Samples

The cerebral cortex and hippocampal CA1 region of SHC3d control and R3d ischemic animals were homogenized 1:5 (*w*/*v*) in ice-cold buffer A (20 mM Tris-HCl, pH 7.5, 140 mM potassium chloride, 5 mM magnesium acetate, 1 mM dithiothreitol (DTT), 2 mM benzamidine, 1 mM EDTA, 2 mM EGTA, 10 µg/mL pepstatin A, leupeptin, and antipain, 20 mM sodium β-glycerophosphate; 20 mM sodium molybdate and 0.2 mM sodium orthovanadate) as previously described [31,51]. The tissue homogenate from each animal was centrifuged at 12,000× *g* for 15 min at 4 °C, and the postmitochondrial supernatant (PMS) was collected. All procedures were performed at 4 °C. Protein concentration was determined by Bradford assay (Bio-Rad). PMS samples corresponding to each animal were separately kept at −80 °C until used and thawed only once just before use. The experimental sample groups were: SHC3dC and SHC3dCA1, samples from the 3-day sham-control animals of the cerebral cortex and hippocampal CA1 region, respectively; R3dC and R3dCA1, samples from 3-day reperfusion-ischemic animals of the cerebral cortex and hippocampal CA1 region, respectively.

### 4.4. Immunoprecipitation

PMS samples (100–1200 µg of protein) from each experimental condition were preclarified, incubating (50–100 µL) with Protein G-Agarose 4 (25 µL; 50% slurry, *v*/*v* in buffer A; ABT) for 1 h at 4 °C on a rotary shaker and then centrifuged at 16,000× *g* for 5 min to remove nonspecifically attached proteins. Supernatants were then incubated with primary anti-eIF4E-BP2 or anti-DRP2 or anti-eIF4E antibody (1–9 µg) overnight at 4 °C and then further incubated with Protein G-Agarose 4 (25 µL; 50% slurry, *v*/*v* in buffer A) for 1 h at 4 °C on a rotary shaker. The immunoprecipitates were recovered by centrifugation at 2500× *g* for 5 min and washed and centrifuged successively three times in buffer A without DTT. Finally, immunoprecipitated proteins were eluted with a loading buffer (60 mM Tris-HCl, pH 6.8; 3% SDS; 2% β-mercaptoethanol; 5% glycerol; 0.0083% bromophenol blue) for one-dimensional SDS-PAGE analysis [21], or with 8.5 M urea for 30 min and centrifugated at 16,000× *g* for 10 min at room temperature for two-dimensional gel electrophoresis (see below). Control experiments were performed in parallel using samples without antibody incubation.

### 4.5. Two-Dimensional Fluorescence Difference in Gel Electrophoresis

The 4E-BP2 immunoprecipitates from each different experimental condition (SHC3dC, SHC3dCA1, R3dC, and R3dCA1) were analyzed by two-dimensional (2D) fluorescence difference in gel electrophoresis (DIGE). A balanced mixture of 4E-BP2 immunoprecipitates from all of the sample groups was used as the internal standard for quantitative comparisons [52], as previously described [53]. Samples of 4E-BP2 immunoprecipitates from the control or ischemic groups were labelled with Cy5 or Cy3 dyes and the internal standard mixture was labelled with Cy2 dye, according to standard CyDye DIGE protocols from GE Healthcare, and labelled samples were kept at −80 °C until use. Prior to 2D DIGE, samples were pairwise combined and pooled with the internal standard and added up to 1% β-mercaptoethanol. First-dimensional isoelectric focusing (IEF) was prepared on immobilized strips (pH 3–10) (Bio-Rad) on a flatbed Ettan IPGphor 3 (GE Healthcare). Strips were immersed in a rehydration buffer (8 M urea, 2% CHAPS, 97 mM DeStreak, 0.2% ampholyte pH 3–10, and 0.001% bromophenol blue) for 16 h and labelled mixtures were applied using a loading cup near to the acidic end of the strip. After focusing with an overall voltage of 10 kV, strips were incubated for 15 min in an equilibration buffer (75 mM Tris–HCl pH 8.8, 6 M urea, 30% glycerol, 2% SDS, and 0.004% bromophenol blue). Then, to conclude the second-dimension process, the focused samples were loaded onto SDS-PAGE minigels (12% acrylamide, 2.6% cross linking) with an IEF strip as a stacking gel [21,53]. The gels were scanned on a Typhoon 9400 (GE Healthcare) fluorescence imager at 500 V and high pixel resolution (25 μm/pixel) in the area of interest. Cy-tagged images were scanned using a 535 nm laser and 650 BP30 emission filter for Cy3 detection, a 633 nm laser, and 680 BP30 emission filter for Cy5, and a 488 nm laser and 570 BP30 emission filter for Cy2. Protein markers (range: 12–225 kDa) and pI standards (range: 3–10) (Cytiva), were used to calculate the apparent molecular mass and pI. Since the protein content in the 4E-BP2 immunoprecipitates was very low, PMS samples from 4 different animals of each experimental condition were pooled in order to obtain enough amount of protein in the 4E-BP2 immunoprecipitates for the 2D DIGE procedure. Thus, four independent 4E-BP2 immunoprecipitates of cortical and CA1 samples from 16 control animals (SHC3dC and SHC3dCA1 samples, respectively), and the other four 4E-BP2 immunoprecipitated samples of these brain regions from 16 ischemic animals (R3dC and R3dCA1 samples, respectively), were labelled with Cy3 or Cy5 dyes and the different experimental groups (*n* = 4) were combined by pairs in eight computer-assisted combinations to compare all the experimental conditions with each other and resolved by 2D DIGE. DIGE experiments were quantified for biological variation analysis (BVA) between experimental groups (SHC3dC, SHC3dCA1, R3dC, and R3dCA1). For this, scanned DIGE fluorescence images were analyzed for protein abundance variations of DRP2 in 4E-BP2 immunoprecipitates and differentially detected protein spots between experimental groups were quantified using PDQuest 7.4 software (BioRad).

### 4.6. MALDI-TOF Mass Spectrometry

Two-dimensional DIGE gels were used for protein identification by MALDI-TOF MS. Protein spots (gel pieces) were manually excised from the gels and processed for in-gel digestion, performed as described by Shevchenko et al. with minor modifications [22,54]. Supernatants of digested samples were collected for peptide mass-fingerprinting (PMF) analysis by MALDI-TOF MS (Autoflex III Bruker Daltonics, Bremen, Germany) and protein identification as previously described [22,55]. Additionally, when available and for the confirmation of protein identity, peptide fragmentation was performed by MS in tandem with MALDI LIFT-TOF/TOF [56]. MS data from PMFs and MS/MS data from the LIFT TOF/TOF spectra were searched in the SwissProt database using the Mascot database search algorithm (Matrix Science, London, UK) for protein identification. Only one missed tryptic cleavage was allowed and a mass accuracy of 100 ppm was used for mass searches. MALDI-TOF MS and LIFT TOF/TOF spectra are shown in the Supplementary Materials (Figures S3 and S4).

### 4.7. Western Blot

PMS samples (20 μg) or immunoprecipitates (100 μg) were analyzed by one-dimensional SDS-PAGE (12% acrylamide, 2.6% cross-linking). Proteins were transferred onto the PVDF membranes (Cytiva) and incubated in blocking agent (2% Prime Blocking, Cytiva) in 0.1 M phosphate-buffered saline (PBS), pH 7.4 and 0.05% Tween, for 1 h at room temperature. The membranes were then incubated with the primary antibody in blocking solution overnight at 4 °C, washed three times in PBS 0.05% Tween (10 min each), and incubated with peroxidase-conjugated antimouse, antirabbit, or antigoat IgG antibody for 1 h at room temperature. Blots were developed with ECL-Prime, ECL-Select (Cytiva), or Clarity ECL reagent (Bio-Rad). To avoid a potential cross reactivity between the mouse monoclonal and goat polyclonal anti-DRP2 antibodies, protein levels obtained for each antibody were analyzed on independent blots. Quantification was carried out by Quantity One 4.6 software (Bio-Rad). Protein markers (range, 12–225 kDa) (GE Healthcare) were used to calculate the apparent molecular mass.

### 4.8. Statistical Analysis

Data are represented in arbitrary units (A.U.) or relative to reference protein levels (ratios) and expressed as mean ± SE. Statistical significance between experimental groups was determined by a one-way ANOVA test and, when significant, was followed by the Newman–Keuls post-test for multiple group comparisons, or Student’s *t* test for comparisons between the cerebral cortex and CA1 region. All statistical analyses were performed with Prism 5.0 software (GraphPad Software) and the statistical significance level was set at α = 0.05.

## 5. Conclusions

Different isoforms of DRP2 emerge as novel modulators of the 4EBP2–eIF4E complex, suggesting a phosphorylation regulation of DRP2 with consequences on translational rates that could infer brain-tissue survival attributes. Our results make us aware that novel protein–protein interactions may be important regulators in cellular processes such as translation regulation. The differential DRP2 isoforms in brain tissue reported here are certainly findings that require further studies in order to confirm the DRP2-phosphorylation-dependent mechanism involved in the regulation of 4E-BP2 and eIF4E in ischemia reperfusion. Advances in the protein interactions of these critical mechanisms will surely provide greater insight into the role of these proteins in brain-tissue survival and may represent a potential therapeutic approach for the treatment of ischemic stroke. This report suggests that DRP2-phosphorylation-dependent protein-interaction changes appear to be pathological events in cerebral ischemia. Inhibiting DRP2 phosphorylation by developing molecules able to manipulate the post-translational modification state of DRP2 would contribute to neuroprotection and neurorepair in patients after acute ischemic stroke. Further analysis and research of DRP2 as a key protein in the neuroprotection mechanism in cerebral ischemia could constitute a possible target for therapeutic action.

## Figures and Tables

**Table 1:** DRP2 isoforms identified by MALDI-TOF MS in 2D DIGE experiments from 4E-BP2 immunoprecipitates.

Spot No.	Protein Identity/Synonyms ^a^	Accession No. ^b^	Gene Name	Molecular Mass (MM) (kDa)	Apparent MM (kDa)	pI	Apparent pI	Protein Score ^c^	Coverage (%)	LIFT (Score) ^d^
4.1	DRP2 /CRMP2 /TOAD64	P47942	Dpysl2	62.3	66.3	6.0	6.4	199	41	1296.7 (65)
4.2					66.3		6.3	128	33	2070.9 (75)
4.3					66.3		6.2	136	40	2169.0 (63)
3.1					66.5		5.8	79	24	2377.2 (73)
3.2					68.2		5.7	116	38	908.5 (35)

^a^ Abbreviations: DRP2, dihydropyrimidinase-related protein 2; CRMP2, collapsin response mediator protein 2; TOAD64, turned on after division 64 kDa protein. ^b^ Accession number in the UniProt database (https://www.uniprot.org/, accessed on 13 February 2023). ^c^ Protein scores > 51 were significant (*p* < 0.05) by peptide mass fingerprint in the Mascot database search algorithm (Matrix Science, London, UK, http://www.matrixscience.com/, accessed on 13 February 2023). ^d^ MALDI LIFT-TOF/TOF identification mode; scores > 24 were significant (*p* < 0.05) by MS/MS ions search performed in Mascot; the fragmented parental peptide and the score (in parenthesis) are indicated.

## Data Availability

The data presented in this study are available in the article.

## Supplementary Materials

The following supporting information can be downloaded at: https://www.mdpi.com/article/10.3390/ijms24098246/s1.

## References

[1] de Lecinana M.A., Diez-Tejedor E., Gutierrez M., Guerrero S., Carceller F., Roda J.M. (2005). New goals in ischemic stroke therapy: The experimental approach--harmonizing science with practice. Cerebrovasc. Dis..

[2] Chien S., Kandel E.R., Schwartz J.H. (1985). Cerebral Blood Flow and Metabolism. Principles of Neural Science.

[3] Lipton P. (1999). Ischemic cell death in brain neurons. Physiol. Rev..

[4] White B.C., Sullivan J.M., DeGracia D.J., O’Neil B.J., Neumar R.W., Grossman L.I., Rafols J.A., Krause G.S. (2000). Brain ischemia and reperfusion: Molecular mechanisms of neuronal injury. J. Neurol. Sci..

[5] Hara M.R., Snyder S.H. (2007). Cell signaling and neuronal death. Annu. Rev. Pharmacol. Toxicol..

[6] Jin R., Liu L., Zhang S., Nanda A., Li G. (2013). Role of inflammation and its mediators in acute ischemic stroke. J. Cardiovasc. Transl. Res..

[7] Dienel G.A., Pulsinelli W.A., Duffy T.E. (1980). Regional protein synthesis in rat brain following acute hemispheric ischemia. J. Neurochem..

[8] Hossmann K.A. (1993). Disturbances of cerebral protein synthesis and ischemic cell death. Prog. Brain Res..

[9] Kirino T. (2000). Delayed neuronal death. Neuropathology.

[10] Thilmann R., Xie Y., Kleihues P., Kiessling M. (1986). Persistent inhibition of protein synthesis precedes delayed neuronal death in postischemic gerbil hippocampus. Acta Neuropathol..

[11] DeGracia D.J., Hu B.R. (2007). Irreversible translation arrest in the reperfused brain. J. Cereb. Blood Flow Metab..

[12] Hossmann K.A. (2008). Cerebral ischemia: Models, methods and outcomes. Neuropharmacology.

[13] Pulsinelli W.A., Brierley J.B., Plum F. (1982). Temporal profile of neuronal damage in a model of transient forebrain ischemia. Ann. Neurol..

[14] Harukuni I., Bhardwaj A. (2006). Mechanisms of brain injury after global cerebral ischemia. Neurol. Clin..

[15] Gingras A.C., Raught B., Sonenberg N. (2004). mTOR signaling to translation. Curr. Top. Microbiol. Immunol..

[16] Jackson R.J., Hellen C.U., Pestova T.V. (2010). The mechanism of eukaryotic translation initiation and principles of its regulation. Nat. Rev. Mol. Cell Biol..

[17] Haghighat A., Mader S., Pause A., Sonenberg N. (1995). Repression of cap-dependent translation by 4E-binding protein 1: Competition with p220 for binding to eukaryotic initiation factor-4E. EMBO J..

[18] Svitkin Y.V., Herdy B., Costa-Mattioli M., Gingras A.C., Raught B., Sonenberg N. (2005). Eukaryotic translation initiation factor 4E availability controls the switch between cap-dependent and internal ribosomal entry site-mediated translation. Mol. Cell. Biol..

[19] Tsukiyama-Kohara K., Vidal S.M., Gingras A.C., Glover T.W., Hanash S.M., Heng H., Sonenberg N. (1996). Tissue distribution, genomic structure, and chromosome mapping of mouse and human eukaryotic initiation factor 4E-binding proteins 1 and 2. Genomics.

[20] Ayuso M.I., Martinez-Alonso E., Cid C., Alonso de Lecinana M., Alcazar A. (2013). The translational repressor eIF4E-binding protein 2 (4E-BP2) correlates with selective delayed neuronal death after ischemia. J. Cereb. Blood Flow Metab..

[21] Ayuso M.I., Martinez-Alonso E., Salvador N., Bonova P., Regidor I., Alcazar A. (2015). Dissociation of eIF4E-binding protein 2 (4E-BP2) from eIF4E independent of Thr37/Thr46 phosphorylation in the ischemic stress response. PLoS ONE.

[22] Martínez-Alonso E., Guerra-Pérez N., Escobar-Peso A., Regidor I., Masjuan J., Alcázar A. (2021). Differential Association of 4E-BP2-Interacting Proteins Is Related to Selective Delayed Neuronal Death after Ischemia. Int. J. Mol. Sci..

[23] Poon H.F., Vaishnav R.A., Getchell T.V., Getchell M.L., Butterfield D.A. (2006). Quantitative proteomics analysis of differential protein expression and oxidative modification of specific proteins in the brains of old mice. Neurobiol. Aging.

[24] Chen A., Liao W.P., Lu Q., Wong W.S., Wong P.T. (2007). Upregulation of dihydropyrimidinase-related protein 2, spectrin alpha II chain, heat shock cognate protein 70 pseudogene 1 and tropomodulin 2 after focal cerebral ischemia in rats—A proteomics approach. Neurochem. Int..

[25] Zhou Y., Bhatia I., Cai Z., He Q.Y., Cheung P.T., Chiu J.F. (2008). Proteomic analysis of neonatal mouse brain: Evidence for hypoxia- and ischemia-induced dephosphorylation of collapsin response mediator proteins. J. Proteome Res..

[26] Park D.J., Shah F.A., Koh P.O. (2018). Quercetin attenuates neuronal cells damage in a middle cerebral artery occlusion animal model. J. Vet. Med. Sci..

[27] Campos-Martorell M., Salvador N., Monge M., Canals F., Garcia-Bonilla L., Hernandez-Guillamon M., Ayuso M.I., Chacon P., Rosell A., Alcazar A. (2014). Brain proteomics identifies potential simvastatin targets in acute phase of stroke in a rat embolic model. J. Neurochem..

[28] Cuadrado E., Rosell A., Colome N., Hernandez-Guillamon M., Garcia-Berrocoso T., Ribo M., Alcazar A., Ortega-Aznar A., Salinas M., Canals F. (2010). The proteome of human brain after ischemic stroke. J. Neuropathol. Exp. Neurol..

[29] Maurer M.H., Berger C., Wolf M., Fütterer C.D., Feldmann R.E., Schwab S., Kuschinsky W. (2003). The proteome of human brain microdialysate. Proteome Sci..

[30] Gu Y., Hamajima N., Ihara Y. (2000). Neurofibrillary tangle-associated collapsin response mediator protein-2 (CRMP-2) is highly phosphorylated on Thr-509, Ser-518, and Ser-522. Biochemistry.

[31] Martin de la Vega C., Burda J., Nemethova M., Quevedo C., Alcazar A., Martin M.E., Danielisova V., Fando J.L., Salinas M. (2001). Possible mechanisms involved in the down-regulation of translation during transient global ischaemia in the rat brain. Biochem. J..

[32] DeGracia D.J., Kumar R., Owen C.R., Krause G.S., White B.C. (2002). Molecular pathways of protein synthesis inhibition during brain reperfusion: Implications for neuronal survival or death. J. Cereb. Blood Flow Metab..

[33] Garcia-Bonilla L., Cid C., Alcazar A., Burda J., Ayuso I., Salinas M. (2007). Regulatory proteins of eukaryotic initiation factor 2-alpha subunit (eIF2 alpha) phosphatase, under ischemic reperfusion and tolerance. J. Neurochem..

[34] Moutal A., White K.A., Chefdeville A., Laufmann R.N., Vitiello P.F., Feinstein D., Weimer J.M., Khanna R. (2019). Dysregulation of CRMP2 post-translational modifications drive its pathological functions. Mol. Neurobiol..

[35] Nakamura F., Ohshima T., Goshima Y. (2020). Collapsin response mediator proteins: Their biological functions and pathophysiology in neuronal development and regeneration. Front. Cell. Neurosci..

[36] Yoshimura T., Kawano Y., Arimura N., Kawabata S., Kikuchi A., Kaibuchi K. (2005). GSK-3beta regulates phosphorylation of CRMP-2 and neuronal polarity. Cell.

[37] Xing H., Lim Y.A., Chong J.R., Lee J.H., Aarsland D., Ballard C.G., Francis P.T., Chen C.P., Lai M.K. (2016). Increased phosphorylation of collapsin response mediator protein-2 at Thr514 correlates with Î²-amyloid burden and synaptic deficits in Lewy body dementias. Mol. Brain.

[38] Uchida Y., Ohshima T., Sasaki Y., Suzuki H., Yanai S., Yamashita N., Nakamura F., Takei K., Ihara Y., Mikoshiba K. (2005). Semaphorin3A signalling is mediated via sequential Cdk5 and GSK3beta phosphorylation of CRMP2: Implication of common phosphorylating mechanism underlying axon guidance and Alzheimer’s disease. Genes Cells.

[39] Cole A.R., Knebel A., Morrice N.A., Robertson L.A., Irving A.J., Connolly C.N., Sutherland C. (2004). GSK-3 phosphorylation of the Alzheimer epitope within collapsin response mediator proteins regulates axon elongation in primary neurons. J. Biol. Chem..

[40] Cole A.R., Causeret F., Yadirgi G., Hastie C.J., McLauchlan H., McManus E.J., Hernandez F., Eickholt B.J., Nikolic M., Sutherland C. (2006). Distinct priming kinases contribute to differential regulation of collapsin response mediator proteins by glycogen synthase kinase-3 in vivo. J. Biol. Chem..

[41] Arimura N., Ménager C., Kawano Y., Yoshimura T., Kawabata S., Hattori A., Fukata Y., Amano M., Goshima Y., Inagaki M. (2005). Phosphorylation by Rho kinase regulates CRMP-2 activity in growth cones. Mol. Cell. Biol..

[42] Zhu L.Q., Zheng H.Y., Peng C.X., Liu D., Li H.L., Wang Q., Wang J.Z. (2010). Protein phosphatase 2A facilitates axonogenesis by dephosphorylating CRMP2. J. Neurosci..

[43] Gim S.A., Sung J.H., Shah F.A., Kim M.O., Koh P.O. (2013). Ferulic acid regulates the AKT/GSK-3β/CRMP-2 signaling pathway in a middle cerebral artery occlusion animal model. Lab. Anim. Res..

[44] Li X.B., Ding M.X., Ding C.L., Li L.L., Feng J., Yu X.J. (2018). Toll-Like receptor 4 promotes the phosphorylation of CRMP2 via the activation of Rho-kinase in MCAO rats. Mol. Med. Rep..

[45] Xiong T., Tang J., Zhao J., Chen H., Zhao F., Li J., Qu Y., Ferriero D., Mu D. (2012). Involvement of the Akt/GSK-3β/CRMP-2 pathway in axonal injury after hypoxic-ischemic brain damage in neonatal rat. Neuroscience.

[46] Tan M., Ma S., Huang Q., Hu K., Song B., Li M. (2013). GSK-3α/β-mediated phosphorylation of CRMP-2 regulates activity-dependent dendritic growth. J. Neurochem..

[47] He X., Jiang L., Dan Q.Q., Lv Q., Hu Y., Liu J., Wang S.F., Wang T.H. (2017). Bone marrow stromal cells promote neuroplasticity of cerebral ischemic rats via a phosphorylated CRMP2-mediated mechanism. Behav. Brain Res..

[48] Yang L., Lei J.F., Ouyang J.Y., Li M.Z., Zhan Y., Feng X.F., Lu Y., Li M.C., Wang L., Zou H.Y. (2021). Effect of Neurorepair for Motor Functional Recovery Enhanced by Total Saponins From Trillium tschonoskii Maxim. Treatment in a Rat Model of Focal Ischemia. Front. Pharmacol..

[49] Yang L., Li C.Y., Ouyang J.Y., Li M.Z., Zhan Y., Feng X.F., Lu Y., Li M.C., Lei J.F., Zhao T. (2021). Trillium tschonoskii rhizomes’ saponins induces oligodendrogenesis and axonal reorganization for ischemic stroke recovery in rats. J. Ethnopharmacol..

[50] Yamashita N., Ohshima T., Nakamura F., Kolattukudy P., Honnorat J., Mikoshiba K., Goshima Y. (2012). Phosphorylation of CRMP2 (collapsin response mediator protein 2) is involved in proper dendritic field organization. J. Neurosci..

[51] Ayuso M.I., Hernandez-Jimenez M., Martin M.E., Salinas M., Alcazar A. (2010). New hierarchical phosphorylation pathway of the translational repressor eIF4E-binding protein 1 (4E-BP1) in ischemia-reperfusion stress. J. Biol. Chem..

[52] Alban A., David S.O., Bjorkesten L., Andersson C., Sloge E., Lewis S., Currie I. (2003). A novel experimental design for comparative two-dimensional gel analysis: Two-dimensional difference gel electrophoresis incorporating a pooled internal standard. Proteomics.

[53] Cid C., Garcia-Bonilla L., Camafeita E., Burda J., Salinas M., Alcazar A. (2007). Proteomic characterization of protein phosphatase 1 complexes in ischemia-reperfusion and ischemic tolerance. Proteomics.

[54] Shevchenko A., Tomas H., Havlis J., Olsen J.V., Mann M. (2006). In-gel digestion for mass spectrometric characterization of proteins and proteomes. Nat. Protoc..

[55] Martinez-Alonso E., Alcazar P., Camafeita E., Fernandez-Lucas M., Ruiz-Roso G., Alcazar A. (2020). Proteomic analysis of plasma proteins of high-flux haemodialysis and on-line haemodiafiltration patients reveals differences in transthyretin levels related with anaemia. Sci. Rep..

[56] Suckau D., Resemann A., Schuerenberg M., Hufnagel P., Franzen J., Holle A. (2003). A novel MALDI LIFT-TOF/TOF mass spectrometer for proteomics. Anal. Bioanal. Chem..

