# Supplementary material for: Dihydropyrimidinase-Related Protein 2 Is a New Partner in the Binding between 4E-BP2 and eIF4E Related to Neuronal Death after Cerebral Ischemia
Source: Int J Mol Sci. 2023 May 4;24(9):8246. doi: 10.3390/ijms24098246 (PMC10179276; doi:10.3390/ijms24098246)
Supplement: Supplementary file 1 [file ijms-24-08246-s001.zip › ijms-2351501-supplementary.pdf]

Supplementary Material

Dihydropyrimidinase-related protein 2 is a new partner in the binding between 4E-BP2 and eIF4E related to neuronal death after cerebral ischemia

Emma Martínez-Alonso[1,2,*], Alejandro Escobar-Peso[1], Natalia Guerra-Pérez[3], Marcel Roca[1], Jaime Masjuan[4,5] and Alberto Alcázar[1,2,*]

[1] Department of Research, Hospital Universitario Ramón y Cajal, IRYCIS, 28034 Madrid, Spain

[2] Proteomics Unit, Hospital Universitario Ramón y Cajal, IRYCIS, 28034 Madrid, Spain

[3] Department of Genetics, Physiology and Microbiology, Faculty of Biological Sciences, Universidad Complutense de Madrid, 28040 Madrid, Spain

[4] Department of Neurology, Hospital Universitario Ramón y Cajal, IRYCIS, 28034 Madrid, Spain

[5] Department of Neurology, Facultad de Medicina, Universidad de Alcalá, 28871 Alcalá de Henares, Spain

* Correspondence: emma.martinez@hrc.es; alberto.alcazar@hrc.es

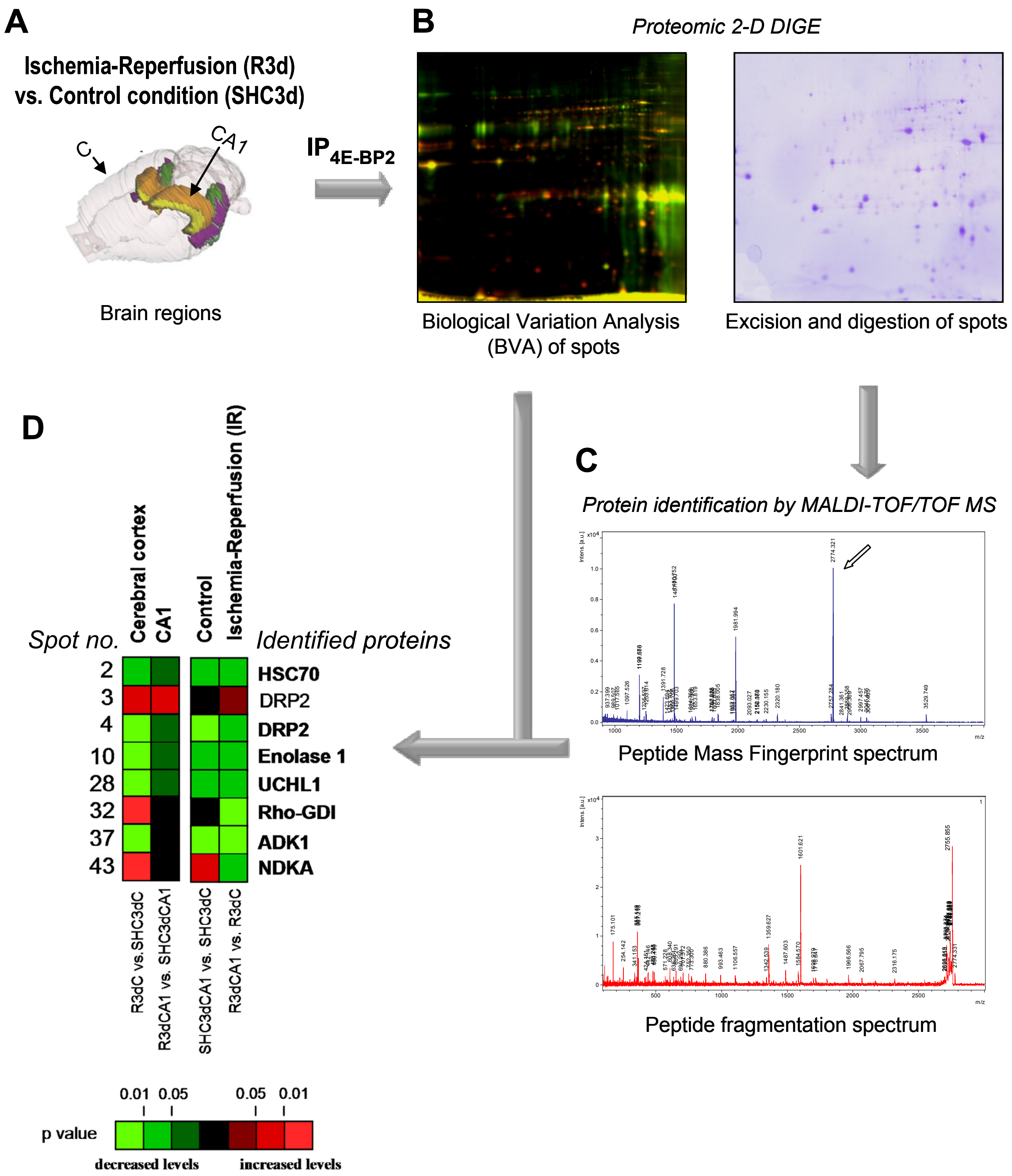
A
Ischemia-Reperfusion (R3d)
vs. Control condition (SHC3d)
C
CA1
Brain regions
IP4E-BP2
B
Proteomic 2-D DIGE
Biological Variation Analysis (BVA) of spots
Excision and digestion of spots
C
Protein identification by MALDI-TOF/TOF MS
Peptide Mass Fingerprint spectrum
Peptide fragmentation spectrum
D
Spot no.
Cerebral cortex
CA1
Control
Ischemia-Reperfusion (IR)
Identified proteins
2 HSC70
3 DRP2
4 DRP2
10 Enolase 1
28 UCHL1
32 Rho-GDI
37 ADK1
43 NDKA
R3dC vs. SHC3dC
R3dCA1 vs. SHC3dCA1
SHC3dCA1 vs. SHC3dC
R3dCA1 vs. R3dC
p value
0.01 0.05 0.05 0.01
decreased levels
increased levels

Figure S1. Experimental procedure for identification of 4E-BP2-interacting proteins in ischemia-reperfusion (IR). Flow chart of the experimental design with a transitory cerebral ischemia animal model, quantification and identification of 4E-BP2-interacting proteins reported in [22]. (**A**) Samples of the cerebral cortex (C-arrow) and hippocampal *cornu ammonis* 1 (CA1) region from ischemic animals after 3 days of reperfusion (R3d) and sham-control animals (SHC3d) were immunoprecipitated with anti-4E-BP2 antibody ($IP_{4E\text{-}BP2}$). (**B**) 4E-BP2 immunoprecipitates from control and ischemic animals were labelled with Cy3 or Cy5 fluorescent dyes and combined in two-dimensional (2-D) fluorescence difference in gel electrophoresis (DIGE) (left image). Detected spots stained with Coomassie blue (right image) were excised and digested to protein identification by mass spectrometry (MS). (**C**) Peptide mass fingerprint and fragmentation spectra obtained by MALDI-TOF/TOF MS were used to protein identification. (**D**) Identified 4E-BP2-interacting proteins were quantified in scanned DIGE gels for biological variation analysis (BVA) to detect the IR response in cortical and CA1 regions compared with control condition (R3dC vs. SHC3dC, and R3dCA1 vs. SHC3dCA1, respectively). Statistical analysis was performed by a post-hoc test after significant ANOVA; colour code represents the statistical significance (*p* value). Abbreviations of 4E-BP2-interacting proteins: HSC70, heat shock 70 kDa protein 8; DRP2, dihydropyrimidinase-related protein 2; UCHL1, ubiquitin carboxyl-terminal hydrolase isozyme L1; Rho-GDI. Rho GDP-dissociation inhibitor 1; ADK1, adenylate kinase isoenzyme 1; NDKA, nucleoside diphosphate kinase A. (Figure **A** has been adapted from Kjonigsen et al., Front Neuroinform 2011, 5:2) (Figures **B**-**D**, have been modified from reference [22])

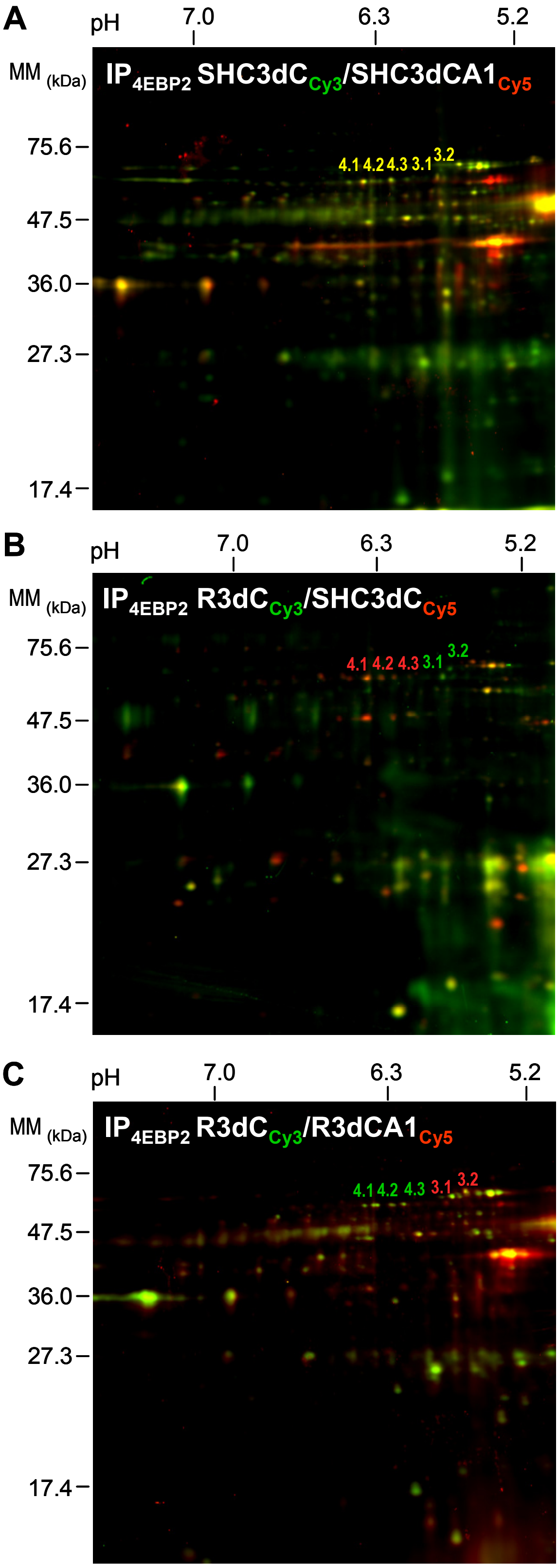

Figure S2. Identification of different DRP2 isoforms associated with 4E-BP2 in vivo. Full 2-D DIGE gels of scanned images showed in Figure 2. Yellow colour is due to the merge of green and red fluorescence labels. The images are representative of 2-D DIGE experiments. Gel images are full original images; the horizontal axis represents pH and the vertical represents axis molecular mass (MM, in kDa).

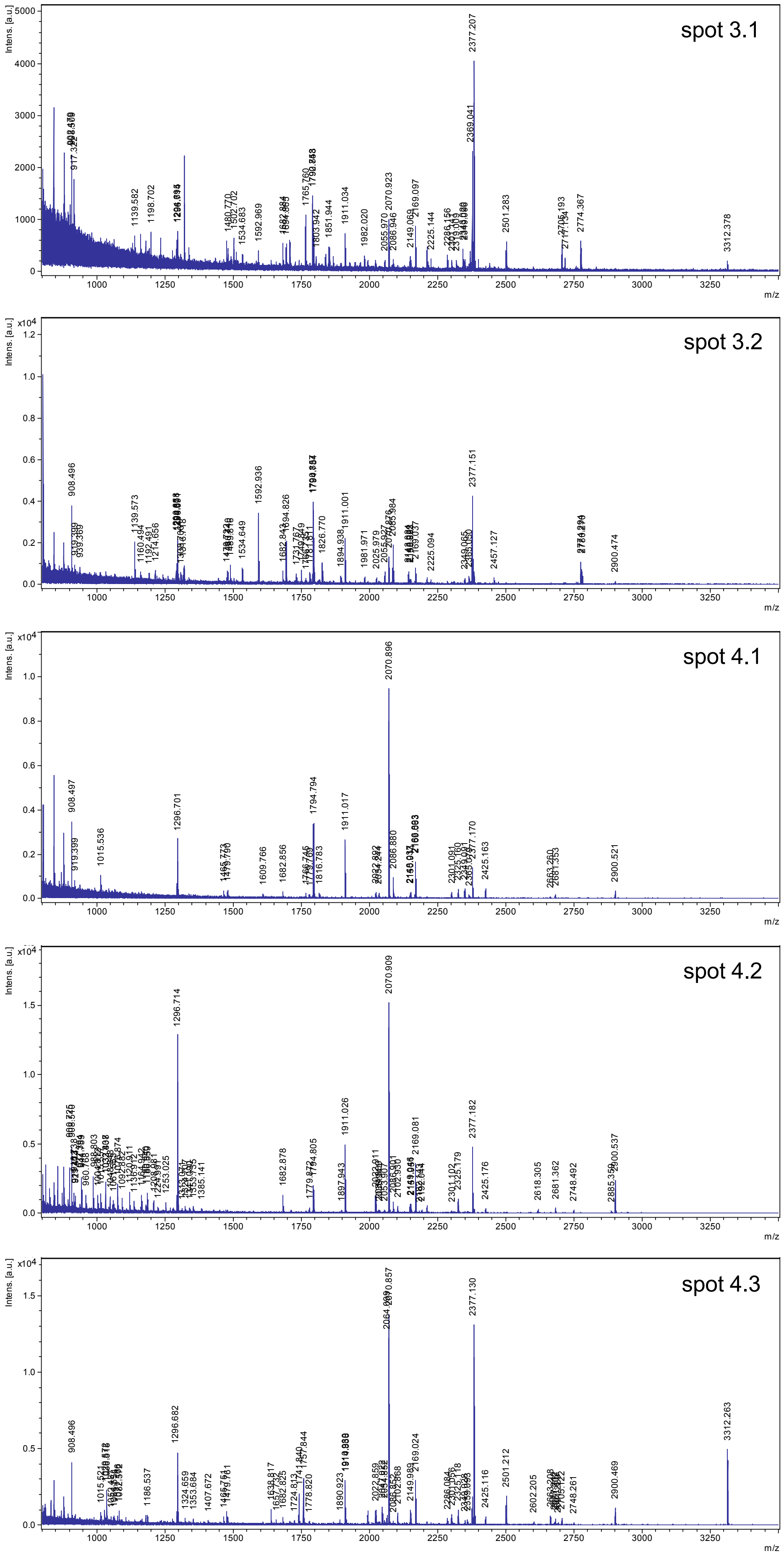

Figure S3. MALDI-TOF MS spectra of DRP2

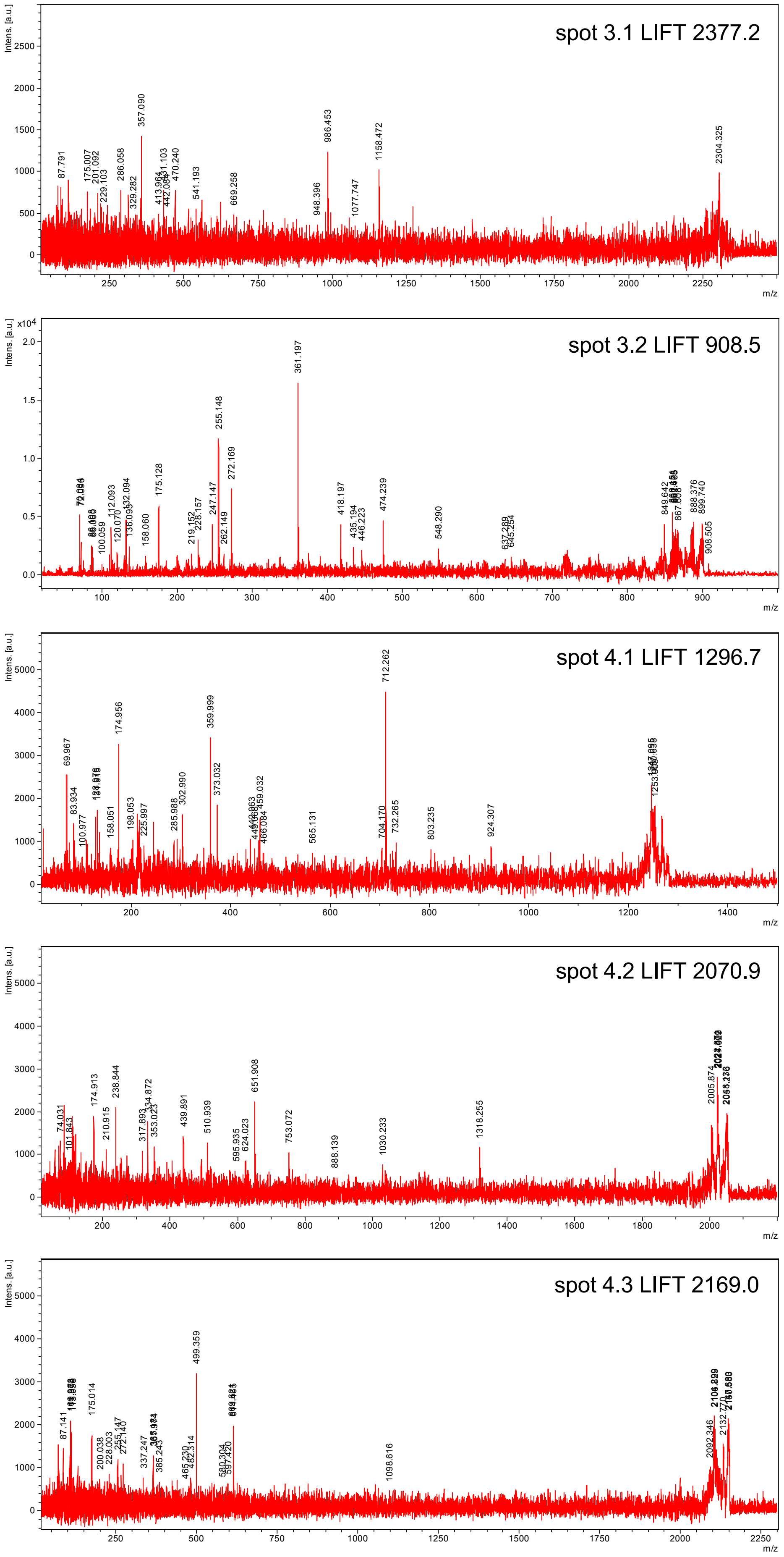

Figure S4. MALDI LIFT-TOF/TOF MS/MS spectra of DRP2
